# Lesion Network Localization of Seizure Freedom following MR-guided  Laser Interstitial Thermal Ablation

**DOI:** 10.1038/s41598-019-55015-y

**Published:** 2019-12-09

**Authors:** Karim Mithani, Alexandre Boutet, Jurgen Germann, Gavin J. B. Elias, Alexander G. Weil, Ashish Shah, Magno Guillen, Byron Bernal, Justin K. Achua, John Ragheb, Elizabeth Donner, Andres M. Lozano, Elysa Widjaja, George M. Ibrahim

**Affiliations:** 1 Faculty of Medicine, University of Toronto, Toronto, ON Canada; 2 University Health Network, Toronto, ON Canada; 3 Joint Department of Medical Imaging, University of Toronto, Toronto, ON Canada; 4 Division of Neurosurgery, CHU-Ste Justine, Université de Montréal, Montréal, Canada; 5 Division of Neurosurgery, Brain Institute, Nicklaus Children’s Hospital, Miami, USA; 6 Department of Radiology, Nicklaus Children’s Hospital, Miami, USA; 7 Division of Neurology, Hospital for Sick Children, Toronto, Canada; 8 Division of Neurosurgery, Department of Surgery, Toronto Western Hospital, University of Toronto, Toronto, Ontario Canada; 9 Department of Diagnostic Imaging, Hospital for Sick Children, Toronto, Canada; 10 Institute of Biomaterials and Biomedical Engineering, University of Toronto, Toronto, Canada; 11 Division of Neurosurgery, Hospital for Sick Children, Department of Surgery, University of Toronto, Toronto, Canada

**Keywords:** Epilepsy, Neural circuits, Epilepsy

## Abstract

Treatment-resistant epilepsy is a common and debilitating neurological condition, for which neurosurgical cure is possible. Despite undergoing nearly identical ablation procedures however, individuals with treatment-resistant epilepsy frequently exhibit heterogeneous outcomes. We hypothesized that treatment response may be related to the brain regions to which MR-guided laser ablation volumes are functionally connected. To test this, we mapped the resting-state functional connectivity of surgical ablations that either resulted in seizure freedom (*N* = 11) or did not result in seizure freedom (*N* = 16) in over 1,000 normative connectomes. There was no difference seizure outcome with respect to the anatomical location of the ablations, and very little overlap between ablation areas was identified using the Dice Index. Ablations that did not result in seizure-freedom were preferentially connected to a number of cortical and subcortical regions, as well as multiple canonical resting-state networks. In contrast, ablations that led to seizure-freedom were more functionally connected to prefrontal cortices. Here, we demonstrate that underlying normative neural circuitry may in part explain heterogenous outcomes following ablation procedures in different brain regions. These findings may ultimately inform target selection for ablative epilepsy surgery based on normative intrinsic connectivity of the targeted volume.

## Introduction

Epilepsy is a common and debilitating neurological illness, affecting nearly 1% of the world’s population^1^. Up to 40% of patients are refractory to medications and may benefit from surgical intervention^2^. It remains unclear why some patients with focal epilepsy undergoing nearly identical resective procedures have different outcomes. For example, patients with temporal lobe epilepsy are significantly more likely to achieve seizure freedom than those with extratemporal epilepsies, by virtue of the location of the lesion, irrespective of the underlying epileptogenic pathology^3–7^.

Recent studies have leveraged a technique known as lesion network mapping to identify connectomes associated with specific conditions and diseases, based on the assumption that heterogeneous lesions in differing locations that result in a shared phenotype can be localized to specific brain networks^8^. That is, disruption of intrinsic brain connectivity at any given node of a network will result in a clinical phenotype that is more attributable to the network itself than the individual brain lesion. This method has been successfully used to map the network basis of a variety of neuropsychiatric conditions, including akinetic mutism, alien limb, and free will, using normative data from healthy populations^8–11^.

Epilepsy may similarly be viewed as a disorder of large-scale brain networks, rather than a particular region of the brain^12–15^. Several studies have suggested that structural, functional, and electrophysiological connectivity of purported epileptogenic zones can portend post-operative seizure outcome^16–25^. Notably, a recent study identified epileptogenic zones and networks on resting state fMRI (rs-fMRI) using Independent Component Analysis that correlate strongly with zones identified with gold-standard intracranial-EEG recordings^26^. Surgical destruction of all rs-fMRI-identified epileptogenic zones resulted in significantly better seizure outcomes than ablation or resection of a single area. Furthermore, post-operative disruption of these rs-fMRI networks by ≥80% – with postoperative normalization of the patient’s rs-fMRI scans was significantly associated with seizure outcomes^27^. In this study, the value of resting-state connectivity was highlighted by the finding that 97% of participants with post-operative rs-fMRI normalization became seizure-free compared to only 3% without rs-fMRI normalization.

Converging findings therefore suggest that the functional connectivity of hypothesized epileptogenic zones can influence their amenability to surgical treatment, and in turn the resulting postoperative outcomes. In the current study, we sought to investigate the relation between normative brain regions to which epileptogenic volumes are connected and seizure outcomes following ablation of these volumes. To achieve this, we characterized differences in intrinsic functional connectivity of surgical ablations that do and do not result in seizure freedom. Focal surgical ablation volumes were seeded in a large normative rs-fMRI data-set to map networks associated with post-surgical seizure-freedom. The relationships between ablation volume location/connectivity and clinical outcomes following Magnetic Resonance guided laser interstitial thermal ablation therapy (MRgLITT) were studied. This procedure results in minimal disruption of brain tissue and allows the quantification of the spatial extent of a therapeutic lesion with high fidelity. Networks associated with seizure response following MRgLITT were analyzed in order to better understand the association between the intrinsic functional connectivity of focal epileptogenic pathologies and outcomes following surgical ablation.

## Methods

### Laser ablations

Twenty-seven children undergoing MRgLITT for refractory epilepsy at three separate institutions – The Hospital for Sick Children (Toronto, Canada), Centre Hospitalier Universitaire Sainte-Justine (Montréal, Canada), and Nicklaus Children’s Hospital (Miami, USA) – were included. At one-year post-operative follow-up, eleven children satisfied the criteria for Engel Class I and were labelled as “seizure-free” (SF), while the remaining sixteen were identified as “not-seizure-free” (NSF). This study involves the use of de-identified retrospective data, complies with the principles outlined in the Declaration of Helsinki, and was approved by the Research Ethics Board at the Hospital for Sick Children.

Details of the MRgLITT procedure have been previously reported^28,29^. Briefly, a Leksell stereotactic frame is placed and a thin-cut CT or CT-angiogram scan is obtained and merged with pre-operative MRI scans to map frame-based coordinates. Depending on the institution, a Medtronic Visualase® (Minneapolis, MN) or Monteris (Neurablate) laser fiber is then guided to the target using a predetermined trajectory. The patient is then brought to an MRI suite where thermal ablations are performed. Ablative doses (65–85% of maximum power) are always preceded by lower energy test doses (35% of maximum power). The procedure is completed under real-time MRI-guidance, ensuring that a complete ablation of the hypothesized epileptogenic region is achieved in each case.

Details regarding image acquisition and processing have also been reported previously^30^. Briefly, high-resolution anatomical T1-weighted images (0.86 × 0.86 × 0.86 mm isotropic voxels) were collected on a 3 T scanner with a single channel transmit/receive head coil (Philips, the Netherlands), before and after the operation during the same anesthetic. The ablation sites for all children were identified and delineated on de-identified post-operative scans using semiautomatic segmentation with ITK-SNAP v1.4.1^31^. Gadolinium contrast was administered in post-operative scans to facilitate robust demarcation of the ablation volume. Segmentations were manually and independently inspected by two authors to ensure accuracy. Importantly, these scans were de-identified prior to segmentation and all individuals involved in generating and reviewing them were blinded to patient outcomes. Next, the segmented ablation masks were linearly then non-linearly transformed to standard space using the MNI-152 standard-space T1-weighted average structural template (2 × 2 × 2 mm). These registrations were completed using the FMRIB’s Linear Image Registration Tool (FLIRT) and then the FMRIB’s Non-linear Image Registration Tool (FNIRT), with nearest neighbor interpolation. Although the ideal method for analyzing neuroimaging data from young adults and children is controversial, the use of the standardized MNI-152 templated and associated atlases is an accepted and widely employed approach^30,32–35^.

In order to measure the extent to which the segmented ablation masks overlapped with each other, Dice indices were computed. Dice indices evaluate the extent of overlap of a binarized volume with any other binarized volume, as follows:

$$Dice\,Index=\frac{2\ast (volume\,of\,overlap)}{(volume\,\#1)+(volume\,\#2)}$$

### Lesion network mapping

**Figure 1:**
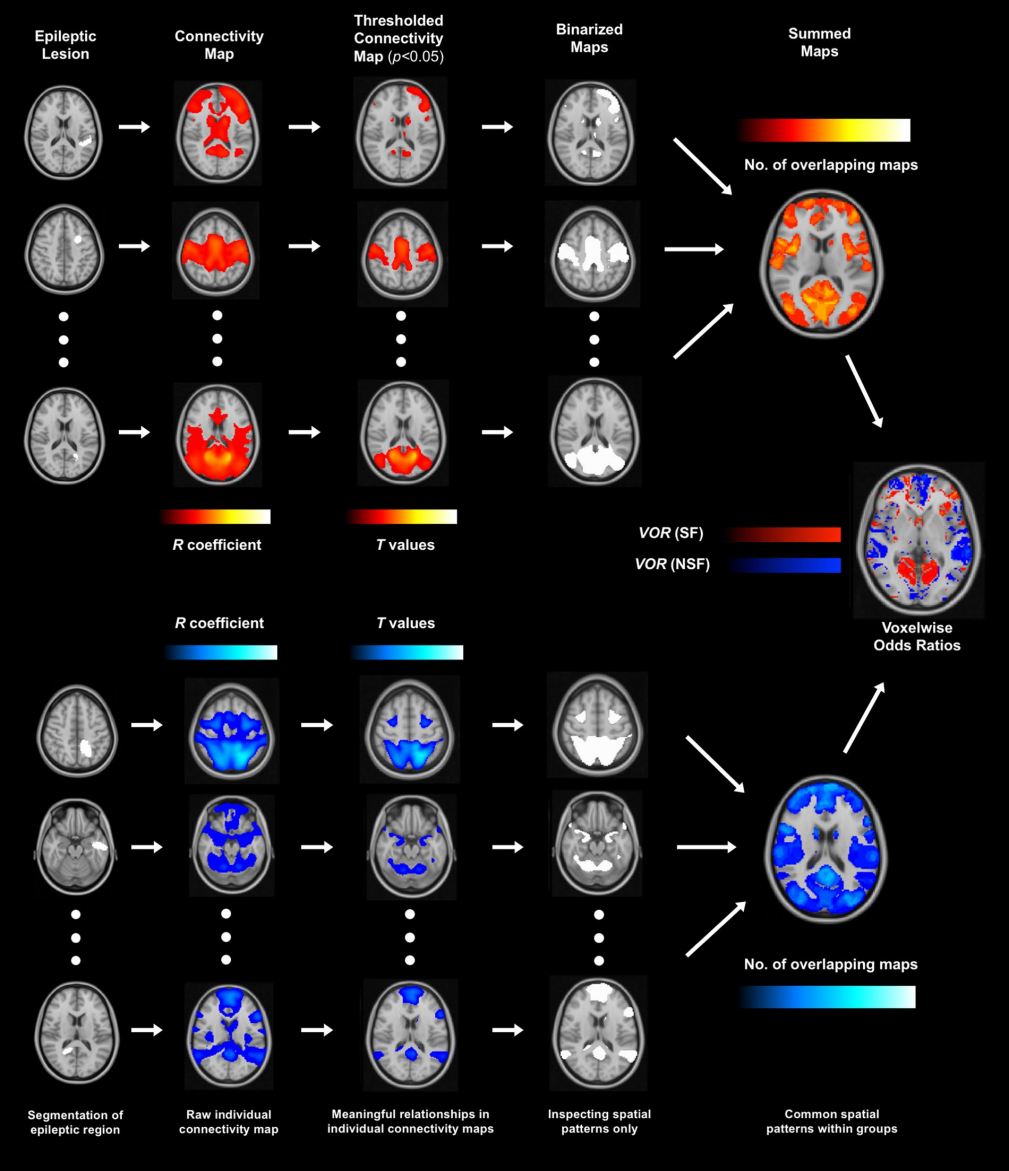
Lesion network mapping methodology and workflow. Segmented lesions were used as seed voxels in a normative dataset of resting-state functional MRI in healthy subjects. Individual connectivity maps were thresholded (*p* < 0.05) to identify meaningful voxels, binarized to inspect spatial patterns, and summed for SF and NSF groups. The summed maps were then compared to yield voxelwise odds ratios.

In order to perform lesion network mapping and identify neural networks connected to the ablation volume that portended seizure outcome, each segmented lesion mask was used to seed a normative rs-fMRI dataset assembled from the 1000 healthy Brain Genomics Superstruct Project (https://dataverse.harvard.edu/dataverse/GSP)^36^, similar to prior analyses^37,38^ (in-house MATLAB script, The MathWorks, Inc., Version R2018a. Natick, MA, USA). A connectivity *r*-map was thus obtained for each individual lesion mask (Fig. 1). To identify brain regions with significant functional connectivity to the ablation volume, these maps were then converted to *t*-maps and thresholded by *t* = 5.1; this corrected for multiple comparisons (Bonferroni corrections) across the whole brain at *p*_*cor*_ < 0.05^38^. The statistically significant thresholded maps were then binarized to obtain meaningful spatial patterns of connectivity associated with each ablation volume. Conscious of the limitations of normative healthy data, this conservative approach ensured that subsequent analyses assessed only the topography of thresholded (*p*_*cor*_ < 0.05) connectivity, rather than the magnitude of correlations themselves, as the latter is known to differ considerably between healthy and epileptic brains^39–41^. The thresholded (p_*cor*_ < 0.05) binarized connectivity masks for each individual were then summed across each group (SF or NSF) to yield overall connectivity patterns associated with lesions that did and did not result in seizure freedom, respectively. A higher voxel value in these summed images indicates a greater number of individual, thresholded connectivity maps overlapping in that particular area of the brain. For example, if the binarized connectivity maps of four separate ablations overlapped at a particular voxel, that voxel would have a value of 4 in the summed image.

These summed connectivity maps for each of the two groups were then used to calculate voxelwise odds ratios (VORs) in order to identify voxels that were more likely associated with lesions that either did or did not result in seizure freedom. VOR maps are generated by comparing the number of lesions that resulted in a particular outcome (e.g. seizure freedom) that are associated with a particular brain region or voxel, to the number of lesions that did not result in that outcome that are also associated with that same region/voxel. This approach has been used in previous lesion mapping studies to identify brain areas associated with risk of a particular outcome, such as central post-stroke pain or adverse effects of focused ultrasound thalamotomy^42,43^.

$$VOR=\frac{{V}_{P}({N}_{C}-{V}_{C})}{{V}_{C}({N}_{P}-{V}_{P})}$$

To calculate the VORs for ablations that resulted in seizure freedom, N_P_ is the number of lesions that resulted in seizure freedom, N_C_ is number of lesions that did not result in seizure freedom, V_P_ is the number of lesions that resulted in seizure freedom with thresholded functional connectivity at a specific voxel, and V_C_ is number of lesions that did not result in seizure freedom with thresholded functional connectivity at a specific voxel. Conversely, to calculate the VORs for ablations that did not result in seizure freedom, N_P_ is the number of lesions that did not result in seizure freedom, N_C_ is number of lesions that did result in seizure freedom, V_P_ is the number of lesions that did not result in seizure freedom with thresholded functional connectivity at a specific voxel, and V_C_ is number of lesions that did result in seizure freedom with thresholded functional connectivity at a specific voxel.

Finally, VOR maps were individually overlaid onto each component of the Automated Anatomical Labelling (AAL) atlas^44^ and the Stanford FIND atlas^45^ to assess mean VORs in specific brain regions and canonical resting-state networks, respectively. The datasets analysed in the current study are available from the corresponding author upon request.

## Results

### Clinical data

Amongst 27 individuals that underwent MRgLITT for intractable epilepsy, 11 achieved seizure freedom (SF) and 16 did not (NSF). The SF and NSF groups did not show statistically significant differences in any key clinical variables, including: sex, age, seizure etiology, pre-operative seizure frequency, or prior epilepsy surgeries (Table 1).

**Table 1:** Clinical and demographic variables of twenty-seven individuals that underwent MRgLITT for intractable epilepsy.

Clinical Variable	Seizure Free (*N* = 11)	Not-Seizure-Free (*N* = 16)	P-value
Sex	6 Females	10 Females	0.71
	5 Males	6 Males	
Age of surgery	13.09 years (range: 8–40)	18.29 years (range: 12–57)	0.14
**Seizure etiology**			
Focal Cortical Dysplasia	2	7	0.49
Tumour	3	2	
Tuberous sclerosis	2	3	
Hypothalamic hamartoma	3	1	
Microgyria	1	2	
Unknown	0	1	
**Lesion location**			
Frontal	3	5	0.88
Parietal	2	3	
Temporal	4	7	
Hypothalamic	2	1	
Lesion volume	3529 mm^3^	4752 mm^3^	0.33

### Lesion characteristics

**Figure 2:**
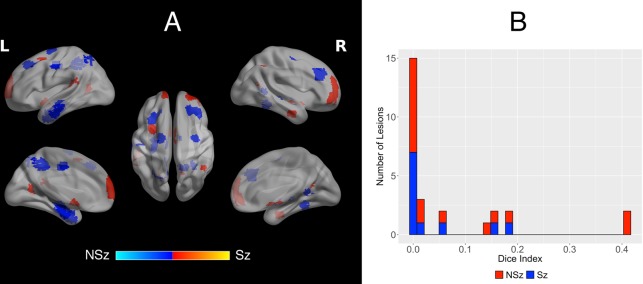
(**A**) Summed ablation masks associated with seizure freedom (SF; red) and non-seizure-freedom (NSF; blue). There is no systematic bias in the localization of ablations in seizure freedom vs. non-freedom groups. The maximum value is 3, in the left hippocampus of the NSF group. (**B**) Dice coefficients quantifying the degree of overlap between lesions in each group. There is no significant difference in average dice index between the two groups (*p* = 0.235).

Ablation targets were identified using multimodal pre-operative investigations aimed at delineating epileptic foci, involving a combination of electrophysiological, metabolic, and structural investigations. The seizure semiologies and epileptogenic zones were variable, with no significant difference between seizure-free and non-seizure-free groups in: temporal vs. extra-temporal localization (*p* = 0.97), mesial (i.e. mesial frontal, mesial temporal, cingulate, or precuneus) vs. non-mesial localization (*p* = 0.46), or ablation volume (*p* = 0.22; Fig. 2A). Specific characteristics of each ablation volume can be found in the Supplementary Material.

The average dice index was 0.0381(SD: 0.0703) for seizure-free ablations, and 0.0883 (SD: 0.141) for non-seizure-free ablations. Other than the left hippocampus where 3 lesions overlapped, this indicates that there was minimal overlap in the location of the individual ablation masks for either group.

### Whole-Brain connectivity patterns

A large, state-of-the-art normative rs-fMRI dataset was leveraged to understand epileptogenic network connectivity associated with seizure outcome. Summed connectivity maps demonstrated diffuse, statistically significant functional connectivity between various regions of the brain and lesions that both did and did not result in seizure freedom (FDR-corrected *p* < 0.05). For both SF and NSF ablations, multiple lesions had regions of overlapping functional connectivity in the prefrontal cortex, cingulate cortex, parietal lobe, and parts of the temporal lobe. NSF ablations demonstrated additional functional connectivity to the bilateral precuneus, hippocampi, temporal lobes and poles, and deep nuclei. Given the significant overlap between functional maps associated with ablations resulting in SF and those associated with NSF, voxelwise odds ratios were computed to compare the likelihood of functional activity in each area being associated with these dichotomous outcomes.

**Figure 3:**
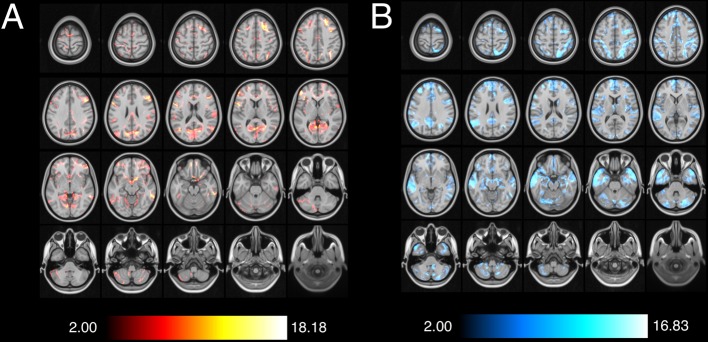
Voxelwise odds ratios of functional activity in various brain regions associated with seizure freedom (**A**) and non-seizure-freedom (**B**).

Ablation targets that resulted in SF were at least twice as likely to be functionally connected to the right orbitofrontal cortex (Mean VOR = 2.11, SD = 1.32, Max VOR = 5.83), left pars opercularis (Mean VOR = 2.23, SD = 1.84, Max VOR = 8.57), and left pars triangularis (Mean VOR = 2.09, SD = 1.67, Max VOR = 8.57) (Table 2). In contrast, ablated regions resulting in NSF were at least twice as likely to be functionally associated with the left orbitofrontal cortex (Mean VOR = 2.18, SD = 1.47, Max VOR = 12.86), left medial frontal gyrus (Mean VOR = 2.47, SD = 1.29, Max VOR = 7.78), left posterior cingulate (Mean VOR = 2.23, SD = 1.12, Max VOR = 3.85), bilateral hippocampi (Mean VOR = 2.08, SD = 1.18, Max VOR = 6.00), bilateral amygdala (Mean VOR = 2.34, SD = 1.26, Max VOR = 6.00), right parietal lobe (Mean VOR = 2.46, SD = 1.53, Max VOR = 12.86), left angular gyrus (Mean VOR = 2.60, SD = 1.81, Max VOR = 9.90), bilateral temporal lobe (Mean VOR = 2.62, SD = 1.80, Max VOR = 13.50), bilateral temporal poles (Mean VOR = 4.18, SD = 1.44, Max VOR = 7.50), and the right cerebellar hemisphere (Mean VOR = 2.19, SD = 1.58, Max VOR = 12.86), amongst others (Table 3). The greatest VOR in the SF group (18.17) was located in the left dorsolateral prefrontal cortex, and the greatest VOR in the NSF group (16.83) was found in the left middle temporal gyrus. Overall, NSF ablations demonstrated a significantly greater spatial distribution of functionally associated areas and larger range of VOR values than SF ablations (Fig. 3).

**Table 2:** Voxelwise odds ratios for key brain regions functionally associated with seizure freedom, based on the AAL atlas.

Region	SF Mean VOR	SD	SF Max VOR	NSF Mean VOR
Right middle frontal gyrus, orbital part	2.11	1.32	5.83	0.73
Left inferior frontal gyrus, pars opercularis	2.30	1.84	8.57	0.78
Right inferior frontal gyrus, pars triangularis	2.10	1.67	8.57	0.87

**Table 3:** Voxelwise odds ratios for key brain regions functionally associated with non-seizure-freedom, based on the AAL atlas.

Region	NSF Mean VOR	SD	NSF Max VOR	SF Mean VOR
Left medial frontal gyrus	2.47	1.29	7.78	0.57
Left medial orbitofrontal cortex	2.18	1.47	12.86	0.61
Left posterior cingulate gyrus	2.23	1.12	3.85	0.64
Left Hippocampus	2.02	1.13	6.00	0.67
Right Hippocampus	2.13	1.23	6.00	0.67
Left Amygdala	2.34	1.31	6.00	0.57
Right Amygdala	2.34	1.21	6.00	0.54
Right superior parietal lobule	2.91	2.04	12.86	0.65
Right inferior parietal lobule	2.00	1.04	7.78	0.70
Left angular gyrus	2.60	1.81	9.90	0.61
Left transverse temporal gyrus (Heschl’s gyrus)	2.06	0.71	3.33	0.55
Left superior temporal gyrus	2.78	1.70	10.00	0.62
Right superior temporal gyrus	2.22	1.61	6.00	0.81
Left middle temporal gyrus	3.02	2.06	13.50	0.64
Left middle temporal pole	4.35	1.35	5.79	0.26
Right middle temporal pole	4.01	1.53	7.50	0.31
Left inferior temporal gyrus	2.47	1.84	7.50	0.87
Right crus I of cerebellum	2.27	1.83	12.86	0.70
Right crus II of cerebellum	2.10	1.16	12.86	0.65
Right lobule IX of cerebellum	2.20	1.76	10.00	0.78
Lobule VII of vermis	2.11	1.49	7.78	0.76

### Resting-State connectomic differences between seizure freedom and Non-freedom

**Figure 4:**
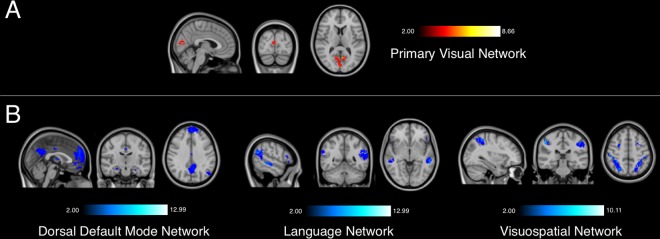
Resting state networks associated with seizure-freedom (**A**) and non-seizure-freedom (**B**). All voxels are at least twice as likely to be functionally associated with each group.

In addition to examining specific brain regions functionally associated with SF and NSF ablations, we also assessed differences between SF and NSF cohorts in terms of ablation volume connectivity to canonical resting-state networks (Table 4). Lesions that do not result in seizure freedom are at least twice as likely to be functionally connected to the dorsal default mode network (DMN), the language network, and visuospatial network (Fig. 4B). In contrast, ablation targets that result in seizure freedom are twice as likely to be functionally connected to only the primary visual network (Fig. 4A).

**Table 4:** Voxelwise odds ratios for canonical functional networks, based on the Stanford FIND Atlas.

Network	NSF Mean VOR	NSF SD	SF Mean VOR	SF SD
Auditory Network	1.51	1.05	1.05	0.65
Basal Ganglia Network	1.35	0.81	1.16	1.01
Left Executive Control Network	1.33	0.84	1.11	0.98
Language Network	2.34	1.66	0.70	0.59
Precuneus Network	1.07	0.71	1.42	1.04
Right Executive Control Network	1.30	0.86	1.29	1.29
Sensorimotor Network	0.95	0.51	1.26	0.47
Visuospatial Network	2.04	1.40	0.84	0.83
Anterior Salience Network	1.78	1.24	0.84	0.60
Dorsal Default Mode Network	2.18	1.11	0.63	0.49
Higher Visual Network	1.02	0.45	1.15	0.44
Posterior Salience Network	1.47	0.89	0.94	0.58
Primary Visual Network	0.55	0.20	2.07	0.92
Ventral Default Mode Network	1.14	1.03	1.41	1.11

## Discussion

Intrinsic brain connectivity is increasingly understood to link common clinical phenotypes that manifest following lesions in different brain regions. Presurgical and resting-state connectivity is also increasingly implicated in seizure-freedom following resective and ablative surgical procedures for epilepsy^16,26,27,30,46–48^. To better understand the network basis of heterogeneous seizure outcomes following MRgLITT, we performed lesion network mapping to identify interconnected brain networks that portend more favorable outcomes. Indeed, we report notable differences in intrinsic connectivity of the epileptogenic foci that correspond to favourable and unfavourable outcomes following ablative epilepsy surgery. Lesions resulting in seizure freedom are more likely to be functionally connected to parts of the prefrontal cortex. In contrast, lesions that do not abolish seizures are preferentially connected to a far greater number of cortical and subcortical regions, as well as multiple canonical resting-state networks.

### Towards a “seizure freedom network”

Given the minimal overlap amongst ablation volumes, our findings suggest that the outcomes of ablative epilepsy surgery may be associated with the resting-state functional connectivity profile – rather than the location – of the targeted region. Epileptogenic foci that are more amenable to surgical ablation are more likely to be connected to a brain network comprising the right orbitofrontal cortex, left pars opercularis, and left pars triangularis, as well as nodes of the primary visual network. Conversely, surgical lesions less likely to abolish seizures show increased connectivity with several cortical and subcortical brain regions – most notably in the temporal lobe – as well as nodes of the dorsal DMN, language network, and visuospatial network. These findings are concordant with prior investigations on the lateralization and location of structural and functional networks in epilepsy^26,27,46^.

The most striking finding in this analysis is the substantial difference in the spatial extent of intrinsic functional connectivity between targeted regions that do and do not result in seizure freedom. Specifically, NSF ablations exhibit robust intrinsic functional connectivity across numerous major cortical and subcortical brain regions, including the nodes of several well-known resting-state networks. In contrast, SF ablations are only functionally associated with a few, specific, relatively well-demarcated foci. Furthermore, NSF ablations also exhibit a considerably larger range of the relative strength of the associations – as quantified by voxelwise odds ratios – than SF ablations. Finally, NSF ablations were functionally associated with deep nuclei, including the caudate and thalami, whereas SF ablations were not. Together, these findings suggest that epileptogenic foci located in areas that are constitutively connected to a wider range of brain regions, and therefore part of a relatively more widespread functional network, are less responsive to ablative surgery. Conversely, epileptogenic foci located in areas with more focused intrinsic functional connectivity are more amenable to ablation. This is consistent with the purpose of MRgLITT epilepsy surgeries, which are aimed at treating focal seizures with precise targets, as well as with prior studies documenting a strong association between the extent of resection of a seizure network and surgical outcomes^27^. A lesion with intrinsically greater spatial extent of functional connectivity is less likely to undergo destruction of its entire functional network, making it less likely to exhibit seizure freedom after MRgLITT ablation.

Interestingly, many of the ablation volumes that did not result in seizure-freedom were functionally connected to temporal structures. Resection of the temporal lobe in temporal lobe epilepsy has the highest likelihood of seizure freedom. Our findings suggest that ablation of extratemporal seizure foci in locations that would normatively be connected to the temporal lobe leads to disappointing outcomes. This may be because the temporal lobe is highly epileptogenic and easily kindled, as evidenced by a number of electrophysiological, metabolic, and molecular studies^49^. Therefore, epileptogenic lesions that are highly functionally connected to parts of the temporal lobe may result in more frequent, severe, and/or intractable seizures and ablating such lesions is evidently less likely to result in seizure freedom. Another potential explanation of this phenomenon is that these patients underwent incomplete or insufficient destruction of the entire functional network connected to the epileptogenic lesion, which is known to be associated with poor surgical outcomes^27^. Both of these explanations emphasize the importance of understanding the functional connectivity of epileptogenic lesions and incorporating this information into surgical planning and targeting.

In addition, our study highlights the importance of canonical resting state networks in propagating epilepsy, which has been emphasized in prior studies^30,50,51^. Specifically, our analysis revealed that epileptogenic lesions associated with the primary visual network are more amenable to ablative surgery, whereas those associated with the language network, visuospatial network, and – perhaps most importantly – the DMN, are not. Functional neuroimaging of patients with numerous forms of drug-resistant epilepsy has consistently identified reduced connectivity and hubness of the DMN in epilepsy compared to healthy controls^52–55^. Furthermore, alterations in DMN activity and connectivity have been reported in focal, idiopathic generalized and absence seizures^39,51,56–58^. In addition, DMN is widely regarded to subserve stimulus-independent emotional processing, self-referential mental activity, and social cognitive functions; this is in keeping with theories that the network may underlie states of altered consciousness seen in certain types of epilepsy^59^. Given the central role of the DMN in intrinsic brain activity, as well as its extensive involvement in mediating various forms of epilepsy, it is possible that increased functional connectivity between its components and epileptogenic lesions is more likely to propagate intractable seizures. Accordingly, lesions in regions that are strongly functionally connected to the DMN would be less amenable to surgical ablation, as demonstrated by our findings.

The other resting-state networks associated with NSF ablations include the language network and the visuospatial network. Notably, several forms of epilepsy – temporal lobe epilepsy in particular – have been associated with impairment of language^60,61^ and visuospatial functioning^62,63^. This again highlights the aforementioned resistant nature of epileptogenic foci that are highly functionally connected to the temporal lobe, even to surgical ablation.

The increased functional connectivity of SF lesions to the primary visual network is of unclear significance, and poses an interesting line of further investigation. It does reinforce the finding that lesions that are amenable to surgical ablation have substantially more focal connectivity than those that are not, given the narrow spatial distribution of the primary visual network. The occipital lobe is also thought to be less epileptogenic than other parts of the brain, such as the temporal lobe, and occipital seizures are relatively less common than many other epilepsy syndromes^64^. Therefore, ablations with greater intrinsic functional connectivity to the primary visual network, as opposed to more widespread networks like the DMN, would be more amenable to surgical ablation, as demonstrated by our findings.

### Implications for clinical practice

Children who undergo nearly identical ablative surgeries for refractory epilepsy demonstrate considerable heterogeneity in outcomes^65–67^. The source of this heterogeneity is not fully elucidated, but remains a critically important question. One potential explanation is under-targeting of functional networks associated with epileptogenic lesions, insufficient destruction of which is associated with a relatively poor surgical outcome^27^. Differences in the intrinsic, normative connectivity of lesions that do and do not respond to surgical ablation can inform the basis of this phenomenon, and further delineate the source of post-surgical htereogeneity after MRgLITT ablation. Characterizing brain connectivity that predisposes to seizure-recurrence following resective or ablative procedures may inform presurgical counseling and potentially guide clinical decision-making. Here, we find that heterogeneity in epilepsy surgery may be explained, at least in part, by the functional networks to which the epileptogenic brain regions are intrinsically connected. This underscores the importance of incorporating functional connectivity into making decisions about surgical candidacy. Future studies investigating the predictive potential of these connectomes can inform the utility of pre-operative functional neuroimaging, either in each individual patient or in publicly available normative databases, in minimally disruptive neurosurgery for epilepsy.

## Limitations and Conclusion

The purpose of ablative or resective epilepsy surgery is to obliterate the epileptogenic zone from the brain, and from any large-scale networks it may be a part of. Accordingly, one interpretation of post-operative seizure recurrence is that the epileptogenic zone was not adequately identified or ablated. However, each of these surgeries was performed with the explicit intention of ablating the entire epileptogenic focus, as verified by real-time MRI-guidance. Given the curative intent of these operations, our findings suggest that in children who are NSF, intrinsic connectivity networks are more likely to be intimately connected to the ablation focus and together comprise the “epileptogenic zone”. Therefore, although post-operative seizure recurrence may be in part a result of inadequate ablation, our analysis strongly implicates differences in intrinsic connectivity of targeted regions in seizure outcomes. Importantly, this is concordant with previous studies demonstrating that insufficient or incomplete destruction of seizure networks connected to an epileptogenic zone, in addition to the lesion itself, are less likely to result in freedom for seizures^27^.

The purpose of this study was to characterize the resting-state functional connectivity of lesions that result in distinct seizure outcomes after ablative surgery, in an attempt to understand clinical heterogeneity. The analyses were all conducted in non-diseased brains, using normative data from 1000 healthy individuals. Functional connectivity between certain brain regions differ in the diseased state, limiting the direct translatability of our findings^68^. However, normative connectivity datasets are derived from a large number of subjects using specialized MRI hardware and acquisition parameters, which may result in a more reliable and reproducible connectivity patterns than native resting-state fMRI. Furthermore, as previously mentioned, we were cognizant of this limitation and opted to use a stringent statistical threshold to establish meaningful connectivity maps. Moreover, we only examined spatial patterns of functional connectivity, rather than attempting to identify individual brain regions associated with seizure outcomes. This is a more conservative approach and is less likely to result in spurious conclusions. Importantly, the purpose of this study was to elucidate the functional connectivity associated with surgical ablations that did and did not respond to seizure freedom, and the results cannot be used to infer causality.

In sum, these results suggest that variability in surgical outcomes can be explained at least in part by the functional connectivity of targeted lesions, even in the absence of differences in gross lesion location, seizure characteristics, and demographic factors. Furthermore, analyzing healthy brains is valuable in identifying true functional connectomes as they are not exposed to disease-related aberrancies and compensatory changes. The current study also pose several new lines of investigation to better understand and clinically translate the connectomics of ablative surgery for epilepsy. Eventually, these and future findings may be useful in predicting whether lesions in specific locations are likely to abolish seizures based on the neuronal networks to which they are intrinsically connected.
